# Sample Reproducibility of Genetic Association Using Different Multimarker TDTs in Genome-Wide Association Studies: Characterization and a New Approach

**DOI:** 10.1371/journal.pone.0029613

**Published:** 2012-02-17

**Authors:** Mara M. Abad-Grau, Nuria Medina-Medina, Rosana Montes-Soldado, Fuencisla Matesanz, Vineet Bafna

**Affiliations:** 1 Departamento de Lenguajes y Sistemas Informáticos, ETS Ingeniera Informática y de Telecomunicaciones - CITIC, Universidad de Granada, Granada, Spain; 2 Instituto de Parasitologa y Biomedicina López Neyra, Consejo Superior de Investigaciones Científicas, Granada, Spain; 3 Department of Computer Science and Engineering, University of California, San Diego, La Jolla, California, United States of America; Johns Hopkins University, United States of America

## Abstract

Multimarker Transmission/Disequilibrium Tests (TDTs) are very robust association tests to population admixture and structure which may be used to identify susceptibility loci in genome-wide association studies. Multimarker TDTs using several markers may increase power by capturing high-degree associations. However, there is also a risk of spurious associations and power reduction due to the increase in degrees of freedom. In this study we show that associations found by tests built on simple null hypotheses are highly reproducible in a second independent data set regardless the number of markers. As a test exhibiting this feature to its maximum, we introduce the *multimarker*  *-Groups TDT (* 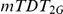 *)*, a test which under the hypothesis of no linkage, asymptotically follows a 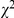 distribution with 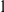 degree of freedom regardless the number of markers. The statistic requires the division of parental haplotypes into two groups: disease susceptibility and disease protective haplotype groups. We assessed the test behavior by performing an extensive simulation study as well as a real-data study using several data sets of two complex diseases. We show that 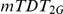 test is highly efficient and it achieves the highest power among all the tests used, even when the null hypothesis is tested in a second independent data set. Therefore, 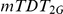 turns out to be a very promising multimarker TDT to perform genome-wide searches for disease susceptibility loci that may be used as a preprocessing step in the construction of more accurate genetic models to predict individual susceptibility to complex diseases.

## Introduction

Current commercially-available genotyping technologies for identifying Single-Nucleotide Polymorphisms (SNPs) are able to scan a few hundred thousands of these binary markers in a single chip array. With such arrays, *in-silico* genome-wide *single nucleotide polymorphisms (SNP)* filtering can be performed as a preprocessing step, before more expensive, molecular-based experimentation, as a way to reduce costs when searching for loci that may be associated to a disease. The most common way of filtering is by performing control-case association studies. However, they are known to inflate type-I errors due to population stratification [1], [2]. An alternative, which is robust to population stratification, is the Transmission/Disequilibrium Test (TDT), a single marker and biallelic test able to detect genetic linkage in the presence of genetic association. Different multimarker generalizations of TDT, such as 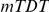 [3], [4], enhance the test by detecting marker interaction, i.e., when a single marker is independent of the trait, but there is association when more than one marker are considered together. This conditional dependence may point out to gene-gene interactions (epistasis), or just to a disease susceptibility gene whose disease allele needs more than one marker to be tagged. TDT is also enhanced by multimarker TDTs when there are no sequenced markers that actually belong to the disease susceptibility locus, but which are in strong linkage disequilibrium (LD) with it [5], [6].

Let us assume that data consist of 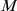 nuclear families with one affected offspring, and that 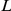 SNPs are genotyped for each family member. As an example, for 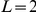, and assuming biallelic SNPs, there will be only 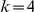 different haplotypes: 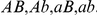 Let us consider a sample 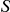 composed of all transmitted and nontransmitted haplotypes whenever parents are heterozygous. Let 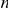 be the sample size, i.e. the number of haplotypes from all heterozygote parents. Thus, the subsample 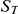 of transmitted haplotypes has 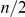 haplotypes, as well as the subsample 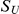 of nontransmitted haplotypes. If all the parents were heterozygous for the genotyped loci, 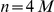 would hold.

In nuclear families with one affected child, there must be a difference between frequencies of nontransmitted and transmitted haplotypes if they are directly associated with the disease, or in linkage with a susceptibility locus. Therefore, at a loci in association with a disease, the most-frequently transmitted haplotypes are disease susceptibility haplotypes. Multimarker TDTs rely on this idea in order to detect linkage in presence of association between a haplotype and a disease susceptibility locus. In contrast to monomarker TDTs, they are more powerful as they are able to detect interaction effects between markers. However, they have an important issue of sample reproducibility. Sample reproducibility refers to the extent to which power reached by a test does not change when the same null hypothesis built using the first data set is used in a second independent data set from the same population. Moreover, the lack of sample reproducibility of multimarker TDTs increases with the number of markers. The reason of this discouraging behavior is because most of them are poorly specific and simultaneously check effects of all the haplotypes found in the data set. For such a generic alternative hypothesis, degrees of freedom (df) strongly increase and very large data sets are required to find consistent associations [7]. Therefore, even if power should increase with haplotype length, the incremental problem of sparse data affects consistency of both power and locus specificity. In practice these tests become inaccurate, except when using one or a couple of SNPs, and their results hardly reproducible in different data sets. Considering the alternative hypothesis as a linkage model composed by sets of haplotypes under the rules of an specific multimarker measure, the number of markers tested together affects model complexity. Therefore, for the same statistic, the higher the number of markers, the larger the data set has to be to detect true associations, i.e. associations in the population, which therefore should also be found in a different data set from the same population. In the very other extreme of only one marker, there will be only two different alleles and very small data sets may be enough for accurate estimators of population models, models which will also replicate in a different data set.

As abovementioned, one example of a multimarker TDT is 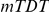 [3], [4], a straightforward extension of 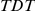 to be used with haplotypes defined as: 
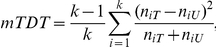
with 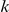 being the number of different alleles/haplotypes and 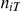, 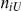 being respectively the number of times an allele/haplotype 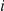 is transmitted and nontransmitted, considering only heterozygous parental genotypes. The measure has a limiting 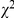 with 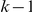 (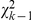) df under no linkage [8]. 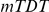 was modified by 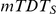 [9], a score method to guarantee that it asymptotically follows an exact 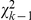 under the null hypothesis of no linkage. Other more recent alternatives are: 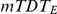 [10], based upon the concept of entropy, whose null distribution is also 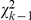 but which reaches lower power than the classic 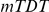 and 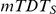 under a wide range of genetic scenearios [6], and 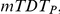 a test which weighs haplotypes by their frequencies and which outperforms 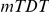 and 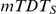 under the ‘common disease-common variant’ (CDCD) hypothesis [6].

Some solutions to reduce df have been proposed, such as grouping haplotypes or using measures based on haplotype similarities [2], [7], [11], [12]. Sometimes, criteria used to select groups may rely on strong assumptions that reduce the power whenever they do not hold. This is the case for 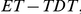 a group-based test that uses a haplotype evolutionary relationship [13] that first requires estimation of a cladogram, which assumes no recurrent disease mutations and no recombination or gene conversion. Perhaps the simplest group-based multimarker TDT is 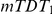 [8], [14], which uses the maximum of the biallelic TDT statistics computed for each haplotype versus all others combined but does not follow a 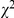 distribution under the null except for haplotypes of only one marker, so that the more markers are used, the larger the false positive rate. The Bonferroni correction is too conservative and other alternatives that do not require unaffordable simulation-based analysis [15] only provide lower and upper bounds to calculate power and type-I errors respectively but are not easily generalized to be used in genome-wide association studies (GWAS) in which power and type-I errors are the two extremes (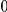 and 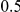 respectively) of an increasing recombination fraction with distance to a disease susceptibility or protective locus. Some similarity-based tests rely also in strong assumptions which reduce the power in a general basis [6]. For example, the Length Contrast Test (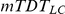) [5], and the Signed Rank Test (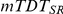) based on 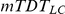 that uses a Wilcoxon score [5], assume that there must be less variation within transmitted haplotypes to affected offspring than within nontransmitted haplotypes [2]. Moreover, the attempts to reduce df yielding to these similarity measures translated as well into an increase in computational complexity. Therefore, the measures are computed by pairwise comparisons between individuals, so that their computational complexity is quadratic on the number of founders, in contrast with most TDT measures, which use sample frequencies and are linear for the number of individuals. For current data sets, like those used in this work which contained over two thousand individuals, this constitutes an important burden when used for genome-wide searching. If the distribution under the null hypothesis is unknown, and has to be estimated using permutations, as it is the case with most similarity and group-based tests [2], [5], [12], [16], [17], the computational time can also increase significantly. Even if computational complexity is linear to the number of permutations, the test is not a practical choice for use in genome-wide association searches.

After showing how state-of-the-art multimarker TDTs reduce sample reproducibility with the increase in the number of markers, our goal was to define a highly powerful, locus specific and computationally feasible multimarker TDT for performing genome-wide association searches which is also highly reproducible when a second data set from the same population is used. We conjectured that reducing df to a minimum regardless to the number of markers should help to reach this goal, and we defined 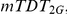 a multimarker TDT that is 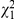 under the null. To achieve this reduction in df, haplotypes are categorized into only two groups: one group represents the disease susceptibility haplotypes and is composed of those haplotypes whose transmission count is higher than their non-transmission count, while the other group represents the protective haplotypes and is composed of those haplotypes that are more frequently nontransmitted. The idea of grouping haplotypes in low and high risk ones was already suggested [14] but no alternative solution was provided to supersede the risk of inflated type-I errors if ad-hoc grouping were performed. In this work we go ahead with this idea and propose a simple alternative approach to ad-hoc grouping, called *holdout*, to avoid the common problem of multiple testing (sample overfitting) in group-based association tests which would yield to inflated type-I errors when more than one marker is used at a time and which becomes very severe for haplotypes with a few markers. Therefore our approach guarantees the statistic is 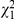 under the null. Under this approach, we randomly divide the data set into two halves, and use one half to choose the two haplotype groups and the other one to infer statistical significance. More complex multisampling approaches such as cross-validation, which divides the data set into at least two folds and obtains a central statistic from the measure obtained by each fold, could be used. However, power may be inflated because dependence between data subsets makes the statistic not to follow a chi square under the null hypothesis of no linkage. We performed simulations in order to compare power, locus specificity and sample reproducibility of 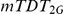 with several state-of-the-art multimarker TDTs. We also tested 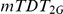 using real data sets comprising family trios with offspring having a complex disease. We showed that 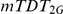 can be used to narrow down regions known to contain some susceptibility loci to multiple sclerosis (MS) and Crohn diseases that are either too wide or undetectable by other multimarker TDTs. We also used the holdout approach with 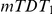, which we have called 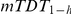, instead of using corrections which tend to over-correct results, such as the Bonferroni correction [18], or which become unaffordable for genome wide scan such as permutation-based corrections [12].

## Results

### The 2-groups multimarker TDT

As abovementioned, 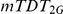 reduces df by further relaxing the small assumptions made in the definition of 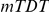 or 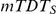. Thus, the test does not assume any fixed number of different haplotypes within the population, as there may always be haplotypes in a population that do not appear in the data set used. It only considers two groups: group 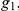 or high-risk group, with all the haplotypes that are most often transmitted to affected individuals, versus group 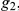 or low-risk group, with all the haplotypes that are most often non transmitted to affected individuals.
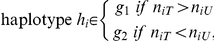
Those haplotypes with the same number of transmitted and nontransmitted counts are not included in any group. Moreover, once the groups are defined, and in order to compute the statistic for a data set, those parental genotypes whose two haplotypes belong to the same group are considered homozygous and are disregarded as all the haplotypes in the same group are collapsed.

Except for only one biallelic marker where there is only one model (two haplotypes), there is always a risk of sample overfitting, i.e. inflated power, which increases with the number of markers as the number of different models also increases. Therefore, there are 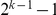 different ways of dividing haplotypes between two disjoint and non-empty groups, with 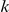 being the number of different haplotypes in the sample. If the same data set were used and no correction were performed, the problem of overfitting would arise: the statistic would be overfitted to that data set, with much larger values than when a different data set were used to infer the groups. Therefore, it would barely be reproducible in a different data set from the same population, with lack of sample reproducibility increasing with the number of markers. If a classical linear multiple testing correction were performed such as the Bonferroni correction, power would strongly decrease, as true association results would be over-corrected [18].

Our solution applies holdout, a very simple multisampling approach: the data set with parental genotypes is divided into two, by default equally sized, data subsets, so that one (the training data set) is used to learn the model and the other (the test data set) to compute the statistic. Therefore, the training data set is used to define the groups, i.e. to assign each haplotype inside the data set to one of the 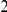 groups, and the counts to compute the statistic are obtained by using only 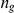 genotypes of the test data set: those heterozygous parental genotypes with one haplotype in each group. To assign a haplotype in the test data set to a group, the following rule is used:
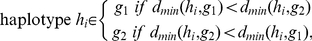
(1)with 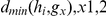 being defined as the distance between 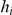 and the haplotype in 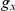 most similar to 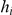. As similarity measure we chose the length similarity measure [5], [12], [19], which equals the largest number of consecutive markers with matching alleles and which is also used in 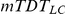 and 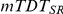 [5].

The 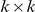 table with haplotype transmissions (one column and row per haplotype), is reduced by 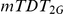 to only two cells in a 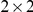 table, with rows representing transmitted group counts and columns representing non transmitted group counts (see Table 1). The first row, second column contains 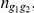 the number of times a heterozygous parent from the test data set with one haplotype in each group transmits the haplotype belonging to 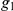 to their offspring and does not transmit the one belonging to 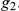 In an equivalent way, the first column, second row contains 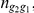 the number of times a heterozygous parent from the test data set with one haplotype in each group transmits the haplotype belonging to 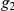 to their offspring and does not transmit the one belonging to 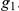 Therefore, counts for each used cell, defined by whether 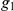 is transmitted (T) and 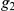 not (U) or the other way around, are computed by summing up the counts of all the genotypes with one haplotype in each group and the same transmission status. Hence, 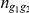 is computed as:
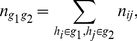
(2)with 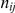 being the number of parents with genotype 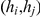 transmitting haplotype 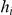 to their offspring. The other count 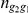 is computed in an equivalent manner.

**Table 1:** The 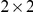 table used by 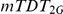.

	Nontransmitted group		
Transmitted group	g1	g2	Total
g1	-	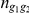	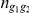
g2	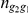	-	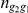
Total	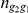	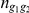	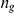

Only those 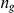 parental genotypes with one haplotype in each group are used by 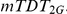 The counts refer to the number of times haplotypes in one group are transmitted by heterozygous parents to their affected offspring.

The statistic is defined as: 
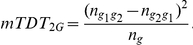


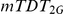
 checks differences in transmissions of group 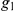 versus group 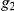, so that it is a McNemar test (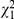) equivalent to the single locus biallelic TDT whenever haplotypes are collapsed into groups and counts were computed by using a different data set. Text S1 shows that 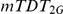 is 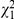 under the null hypothesis of no linkage.

It is straightforward to show that if groups were inferred from the same data set from which the statistic is computed, 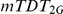 defaults to the usual formula of simple TDT in the case of only one biallelic marker.

Tables 2, 3 and 4 show how to compute 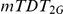 in a simple example with only two biallelic markers. The data set is first divided into two equal-size data subsets (see Table 2). Table 3 left grid shows a 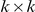 table (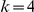) with counts for the training data subset, i.e. the one used to make up groups, using rows to represent transmitted haplotypes and columns to represent nontransmitted haplotypes. As it is shown, the only haplotype in the training data set which is more often non transmitted (56 times) than transmitted (40 times) is 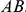 Therefore, group 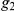 contains only this haplotype. Haplotypes 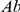 and 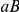 have transmission counts smaller than non-transmission counts so that they make up group 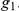 As haplotype 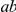 is transmitted as many times as it is non-transmitted, it is not assigned to any group. Table 3 right grid shows a 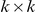 table (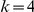) with counts from the test data subset, i.e. the one used to compute the statistic. These counts are used to fill two cells in Table 4, the only two cells in a 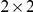 table of group counts used by 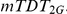 To obtain the counts for Table 4 from Table 3 right grid (test data subset) the haplotypes are first assigned to each group defined by the training data set. Following Equation 1 haplotype 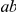 is assigned to the group with the most similar haplotype. As the two most similar haplotypes belongs to group 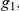 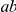 is also assigned to this group.

**Table 2:** An example of parental genotype counts showing transmitted and nontransmitted haplotypes in a training and test data sets of nuclear families and haplotypes of length 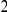 (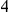 different haplotypes: AB, AB, aB and ab).

Genotype configuration ID	Transmitted haplotype	Nontransmitted haplotype	Counts in Training data set	Counts in Test data set
1	AB	AB	25	30
2	AB	Ab	30	24
3	AB	aB	3	5
4	AB	ab	7	5
5	Ab	AB	37	31
6	Ab	Ab	21	21
7	Ab	aB	6	7
8	Ab	ab	5	4
9	aB	AB	8	9
10	aB	Ab	6	8
11	aB	aB	2	2
12	aB	ab	3	3
13	ab	AB	11	11
14	ab	Ab	3	4
15	ab	aB	1	2
16	ab	ab	0	2
Total parental genotypes			168	168
Total trios			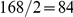	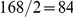

The total number of trios is 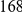 (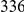 parents) so that half of them (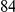 trios, 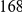 parents) were randomly assigned to the training data set and the others to the test data set. Each row shows counts for a possible configuration (there are 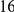 possible configurations for haplotypes of length 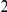) of the transmitted (second column) and nontransmitted (third column) haplotypes in a parental genotype.

**Table 3:** Genotype counts and their transmissions used by 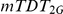.

		Nontransmitted							Nontransmitted				
		haplotype							haplotype				
Transmitted		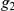	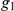				Transmitted		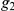	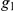			
haplotype		AB	Ab	aB	ab	Total	haplotype		AB	Ab	aB	ab	Total
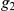	AB	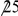	30	3	7	**40**	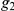	AB	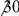	24	5	5	**34**
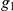	Ab	37	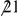	6	5	**48**	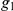	Ab	31	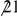	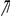	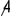	**31**
	aB	8	6	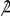	3	**17**		aB	9	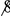	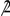	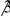	**9**
	ab	11	3	1	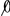	**15**		ab	11	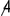	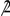	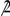	**11**
	Total	**56**	**39**	**10**	**15**	**121**		Total	**51**	**24**	**5**	**5**	**85**

Haplotypes in rows represent those transmitted haplotypes at each genotype. Haplotypes in columns represent those nontransmitted haplotypes at each genotype. Homozygous genotype counts (diagonal) are crossed off the tables as they are not used to compute 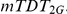 Left grid: genotype counts from the training data set (see Table 2) used to make up groups 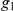 and 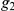 in 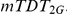 Groups are: 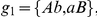 with those haplotypes with 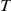 counts larger than 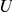 counts (Ab: 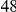 versus 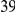 and aB: 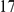 versus 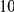) and 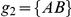 with 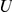 counts larger than 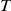 counts (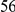 versus 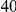). Right grid: genotype counts from the test data set used to compute the statistic. As the length similarity measure is used to assign an haplotype to a group, and the two most similar haplotypes to haplotype 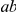 belongs to group 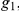 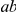 is assigned to 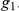 All the haplotypes belonging to the same group are considered of having an equivalent effect and are collapsed. Therefore, parental genotypes in the test data set with haplotypes belonging to the same group are considered as homozygous and not used by 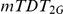 (they are crossed off the table too).

**Table 4:** The 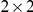 table built by 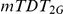: an example.

	Nontransmitted group		
Transmitted			
group	g1: Ab, aB, ab	g2: AB	Total
g1: Ab, aB, ab	-	31+9+11	51
g2: AB	24+5+5	-	34
Total	34	51	85

The table represents group counts, where groups are defined from the training data set, instead of original haplotype counts (see left grid at Table 3). The counts are obtained from the test data set (see those counts not crossed off in Right grid at Table 3). As all the haplotypes in the same group are collapsed, genotypes with both haplotypes in the same group are disregarded. Therefore counts required to compute 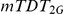 are: 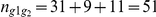 and 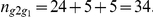

Note that 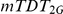 collapses all haplotypes in each group. Therefore, only those individuals with one haplotype at each group 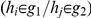 are considered.

### Implementation

The test has been implemented in 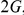 an open source (GPL 2 license) GNU c++ software which can be download from the supplementary website (http://bios.ugr.es/2G).

### Results from simulations

We have performed four sets of simulation studies. The purpose of the first set of simulations was to test sample reproducibility in some state-of-the-art methods. The purpose of the second set of simulations was to show 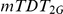 is robust to population stratification and admixture. The purpose of the third set of simulations was to test sample reproducibility of 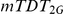 and other tests when used under the holdout approach (see Section Materials and Methods for a detailed explanation about the simulation studies). Finally, the four set of simulations was used to show robustness of 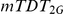 to different proportions of missing haplotypes.

In the first set of simulation results, it can be shown how 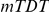 and 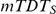 hardly increased power or even reduced it with an increase in the number of markers (window size). It is also shown how they reduced sample reproducibility with an increase in the number of markers as well.

**Figure 1:**
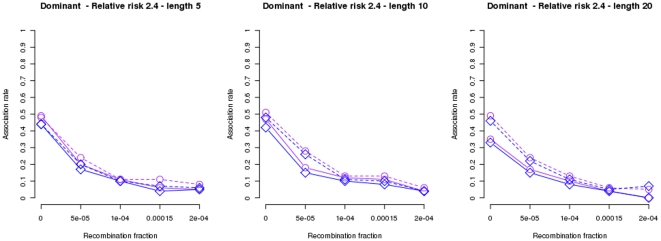
Association rates of 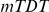 and 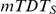 using a second data set to test reproducibility. Results for 100 simulations of 250 family trios as a function of the recombination rate using the dominant and one-locus genetic model and haplotypes of lengths 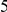 (left plot), 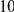 (plot in the middle) and 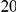 (right plot). A nominal level of 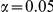 and a relative risk of 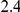 were used for all plots. Results for 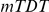 and 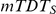 are plotted in purple circles and blue triangles respectively. Dashed lines show results for the data subset (125 trios randomly chosen) used to build the model while solid lines show results for a second data subset (the remaining 125 trios) used to test reproducibility.

Results under the assumption of a dominant genetic model for one disease susceptibility locus and a relative risk of 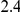 are plotted in Figure 1, which show power (recombination fraction 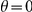) and locus specificity (recombination fraction 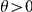) of 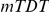 and 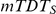 when using a data set to build the hypothesis and compute p values (dashed lines) and when the hypothesis, i.e., a set of haplotypes in association with the disease, is being validated by a second data set (solid lines). The proportion of samples found in association for nominal level 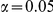 is shown (x-axis). Sample reproducibility, and even power, decreases with the number of markers used: 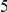 (left plot), 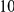 (middle plot) and 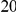 (right plot) due to the problem of sparse data. The same pattern can be observed under a wide range of scenarios (see Figures S1 to S15 at http://bios.ugr.es/2G).

In a second step, we performed simulations to test robustness to population stratification and admixture of 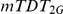 and 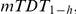 i.e. 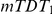 when used under the holdout approach.

Table 5 shows Type I error results for 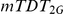 and 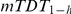 in the presence of population stratification and admixture. Values shown are rates of data sets in which association was found to be statistically significant for nominal level 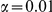 and 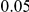 and different haplotype lengths (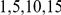 and 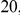 columns 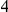 to 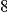 respectively), for all configurations of 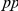 and 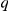 values used (See Section Materials and Methods for a detailed explanation about the different configurations used). It can be seen that values are not significantly different from the nominal values 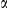, as would be expected in a robust test for population structure and admixture.

**Table 5:** Type I error rates in presence of population stratification and admixture for 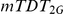 and 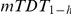.

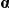	MAFs	pp	l = 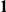	l = 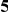	l = 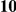	l = 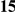	l = 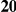
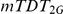							
0.01	0.1	0.5	0.013	0.008	0.008	0.010	0.007
0.01	0.3	0.5	0.014	0.010	0.007	0.010	0.007
0.01	0.5	0.5	0.006	0.009	0.007	0.010	0.009
0.01	0.1	0.75	0.014	0.010	0.009	0.012	0.012
0.01	0.3	0.75	0.017	0.009	0.012	0.015	0.014
0.01	0.5	0.75	0.015	0.010	0.014	0.007	0.009
0.01	0.1	0.833	0.011	0.010	0.008	0.015	0.005
0.01	0.3	0.833	0.013	0.007	0.009	0.012	0.008
0.01	0.5	0.833	0.012	0.007	0.013	0.017	0.007
0.05	0.1	0.5	0.062	0.047	0.043	0.053	0.052
0.05	0.3	0.5	0.063	0.060	0.043	0.048	0.047
0.05	0.5	0.5	0.044	0.055	0.045	0.050	0.048
0.05	0.1	0.75	0.056	0.048	0.056	0.061	0.064
0.05	0.3	0.75	0.061	0.056	0.053	0.061	0.063
0.05	0.5	0.75	0.056	0.050	0.061	0.060	0.058
0.05	0.1	0.833	0.056	0.045	0.046	0.053	0.049
0.05	0.3	0.833	0.060	0.044	0.047	0.061	0.049
0.05	0.5	0.833	0.046	0.044	0.053	0.071	0.056
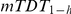							
0.01	0.1	0.5	0.013	0.016	0.009	0.008	0.008
0.01	0.3	0.5	0.014	0.014	0.010	0.008	0.004
0.01	0.5	0.5	0.006	0.017	0.010	0.010	0.015
0.01	0.1	0.75	0.014	0.010	0.012	0.008	0.006
0.01	0.3	0.75	0.017	0.009	0.008	0.008	0.005
0.01	0.5	0.75	0.015	0.007	0.008	0.010	0.009
0.01	0.1	0.833	0.011	0.007	0.011	0.008	0.008
0.01	0.3	0.833	0.013	0.009	0.010	0.010	0.008
0.01	0.5	0.833	0.012	0.008	0.013	0.009	0.008
0.05	0.1	0.5	0.062	0.068	0.051	0.052	0.057
0.05	0.3	0.5	0.062	0.065	0.055	0.048	0.047
0.05	0.5	0.5	0.044	0.068	0.049	0.053	0.065
0.05	0.1	0.75	0.056	0.050	0.052	0.059	0.056
0.05	0.3	0.75	0.061	0.047	0.048	0.065	0.058
0.05	0.5	0.75	0.056	0.058	0.046	0.051	0.056
0.05	0.1	0.833	0.056	0.050	0.050	0.055	0.055
0.05	0.3	0.833	0.060	0.048	0.050	0.058	0.059
0.05	0.5	0.833	0.046	0.050	0.074	0.073	0.061

Results for different minor allele frequencies (MAFs) in the second subpopulation (q) and different proportion of trios from the first subpopulation (pp), obtained by 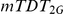 (top half) and 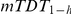 (bottom half) for nominal levels 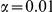 and 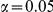 and haplotypes of length 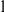, 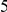, 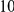, 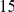 and 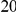 (columns 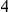 to 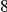 respectively).

In the third set of simulation results, we show how 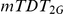 and 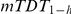 have a good performance in sample reproducibility and how 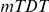 and 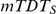 also improve sample reproducibility when using under a holdout approach too, what we called 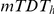 and 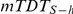 respectively.

In order to check sample reproducibility of 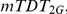 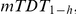 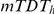 and 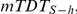 we show p values obtained by the tests in one data set (dashed lines) and by applying the test on a second data set to verify whether associations found in the first data set hold (solid lines). In the second case, the length similarity measure was also used to plug haplotype counts from the second data set into the model obtained from the first data set.

**Figure 2:**
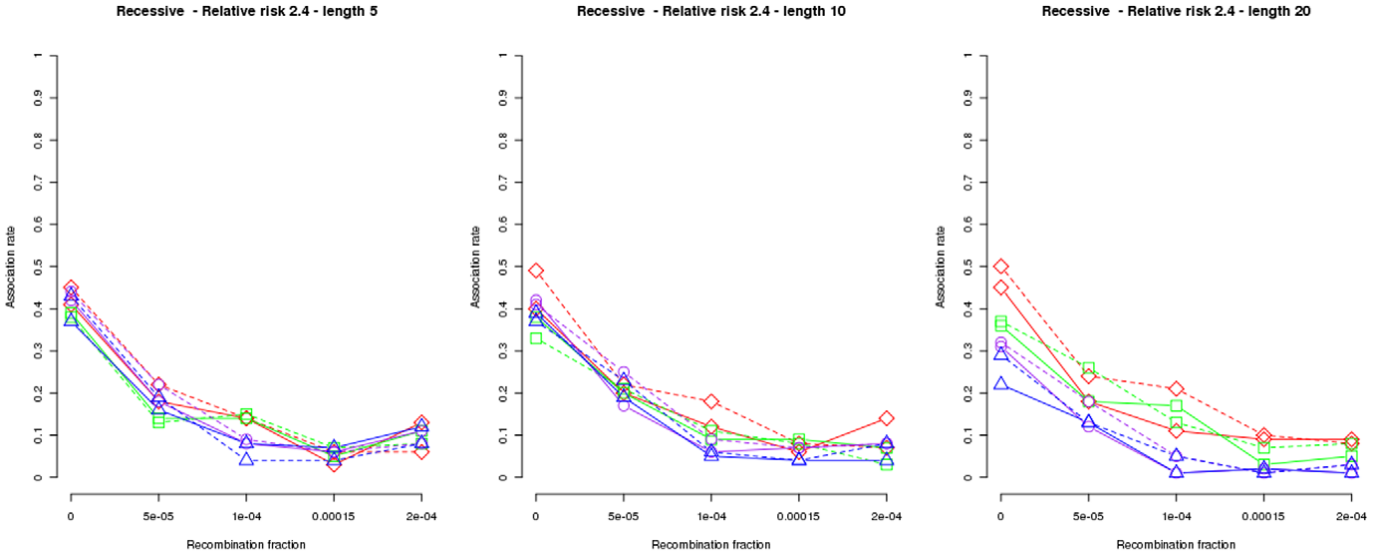
Association rates under the holdout approach using a second data set to test reproducibility. Results for 100 simulations of 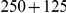 family trios as a function of the recombination rate using the recessive and one-locus genetic model and haplotypes of lengths 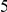 (left plot), 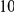 (plot in the middle) and 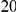 (right plot). A nominal level of 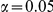 and a relative risk of 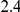 were used for all plots. Results for 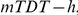 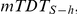 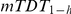 and 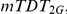 i.e. all tests were applied under the holdout approach, are plotted in purple circles, blue triangles, green squares and red diamonds respectively. Dashed lines show results for a data subset of 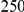 trios randomly chosen while solid lines show results for a second data subset of 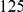 trios used to test reproducibility of the holdout approach.

Results under the assumption of a recessive genetic model for one disease susceptibility locus and a relative risk of 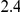 are plotted in Figure 2, to compare power (recombination fraction 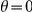) and locus specificity (recombination fraction 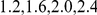 and 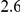) among 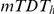 (purple circles), 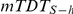 (blue triangles), 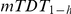 (green squares) and 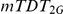 (red diamonds) when the null hypothesis is being validated in a second data set. The proportion of data sets found in association for nominal level 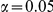 is shown (x-axis). It can be observed how the holdout approach guarantees sample reproducibility, including when used with 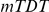 and 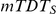, so that differences between dashed and solid lines are smaller compared with those shown in Figure 1.

Moreover, those algorithms with 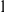 df (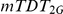 and 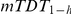) reached the highest power. The differences seem to be more important for smaller relative risks and two disease loci. The same pattern can be observed under a wide range of scenarios (see Figures S16 to S30 at http://bios.ugr.es/2G).

In general, differences among the tests increase with haplotype length. In contrast to 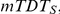 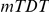 (Figure 1, solid lines) and their holdout versions (Figure 2), power of 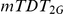 in a second data set increases with the number of markers, even when using 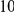 or 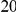 markers. 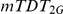 checks a very simple hypothesis: there are differences in transmission frequencies between the two groups of protective and locus susceptibility haplotypes. The reason for a higher power is that, while df do not change with the number of markers, complex associations that cannot be captured with very few markers will be modeled with more markers.


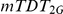
 also outperforms 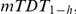 the other test used which has also 1 df. 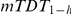 can also be considered a 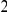-groups test, but there is only one haplotype in one of the groups, and the larger the haplotype the lower the chances of the alternative hypothesis to be confirmed in a second sample. The hypothesis seems to be too simplistic for models with more than one disease locus and power hardly increases when using more than 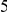 markers.

When the number of markers decreases, the power of the tests tends to converge, down to the situation with only 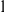 marker, in which 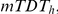 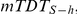 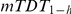 and 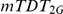 have exactly the same results, as they default to the classic monomarker biallelic 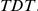 However, when only 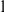 marker is used, power is very low compared with results obtained using longer haplotypes.

Results for the fourth set of simulations are shown in Figure 3 and Figures S31 to S45 at http://bios.ugr.es/2G. These simulations were performed as explained above for the third set of simulations except that association rates (at recombination rate 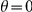) were computed for data sets with 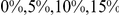 and 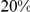 of missing haplotypes.

**Figure 3:**
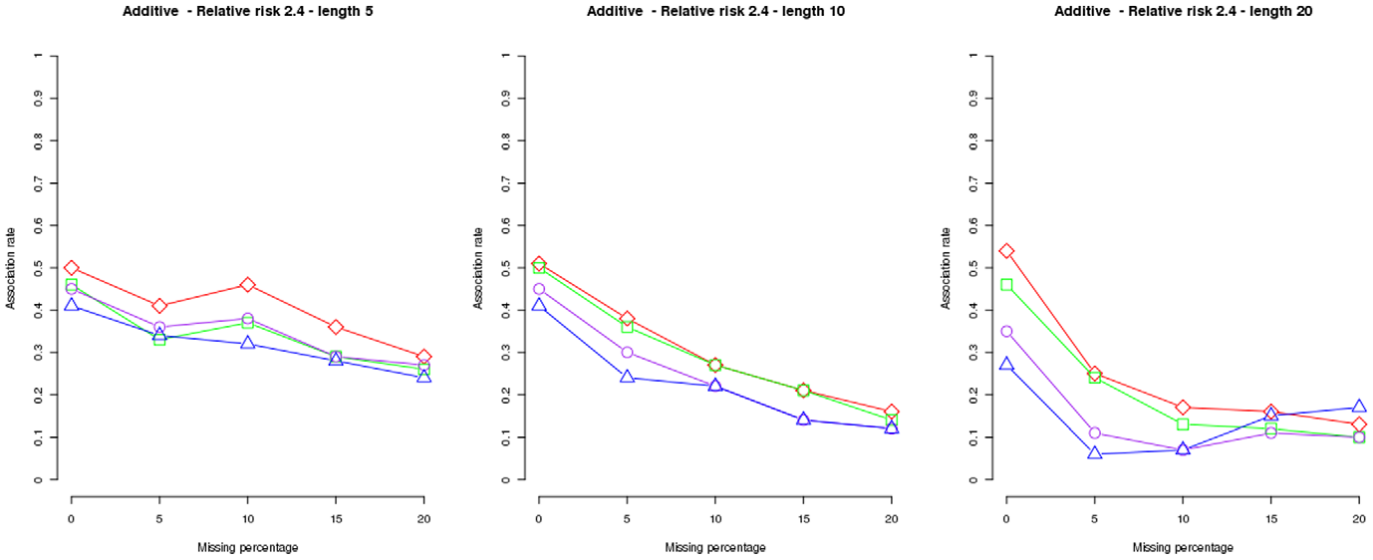
Association rates for different proportions of missing haplotypes. Results for 100 simulations of 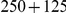 family trios as a function of the proportion of missing haplotypes using the additive and one-locus genetic model and haplotypes of lengths 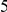 (left plot), 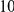 (plot in the middle) and 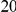 (right plot). A nominal level of 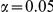 and a relative risk of 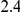 were used for all plots. Results for 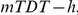 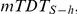 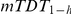 and 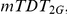 i.e. all tests were applied under the holdout approach, are plotted in purple circles, blue triangles, green squares and red diamonds respectively.

As it can be seen in Figure 3 all the tets used: 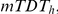 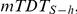 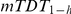 and 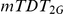 are robust to missing data. Therefore, 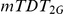 still shows the highest power in data sets with different proportions of missing data.

### Results from real data sets

We tested power and locus specificity using family trio data sets of two complex diseases: Crohn's and MS. We also used trios of unaffected individuals from the International Hapmap Project (IHMP) [20] to measure specificity. We compared power and specificity of 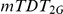 with the most competitive tests considering the wide range of scenarios in our simulations: 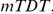 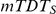 and 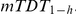


To show results we used sliding windows and *Comparative TDT (CTDT)* [21] maps to plot averaged p values for all the windows (i.e. haplotypes of fixed length starting at a different marker position) covering each marker.

**Figure 4:**
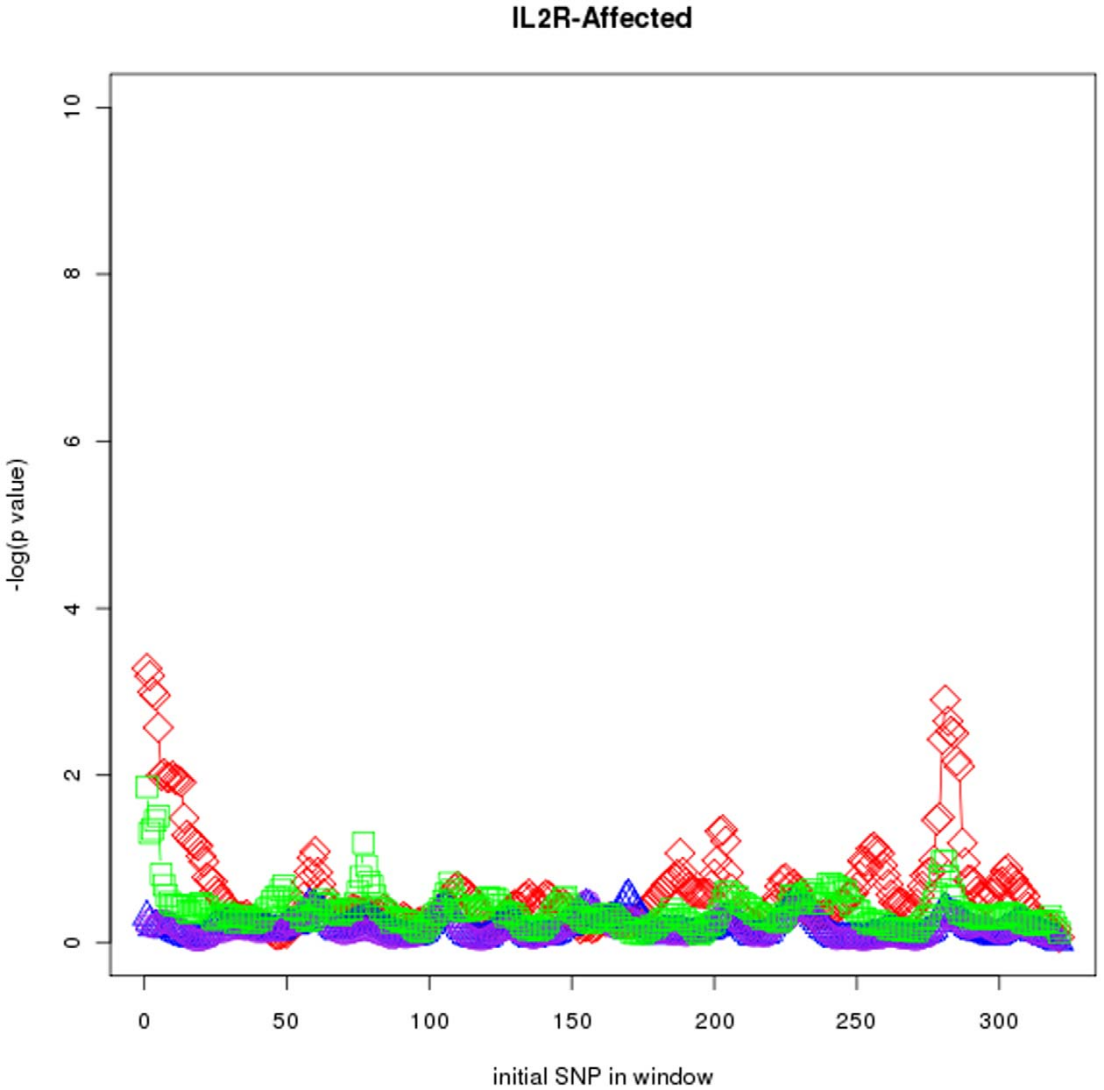
Sliding window maps for the 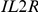-affected data set. Window size is 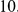 TDTs used were 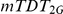 (red diamonds), 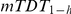 (green squares), 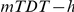 (purple circles) and 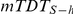 (blue triangles).

**Figure 5:**
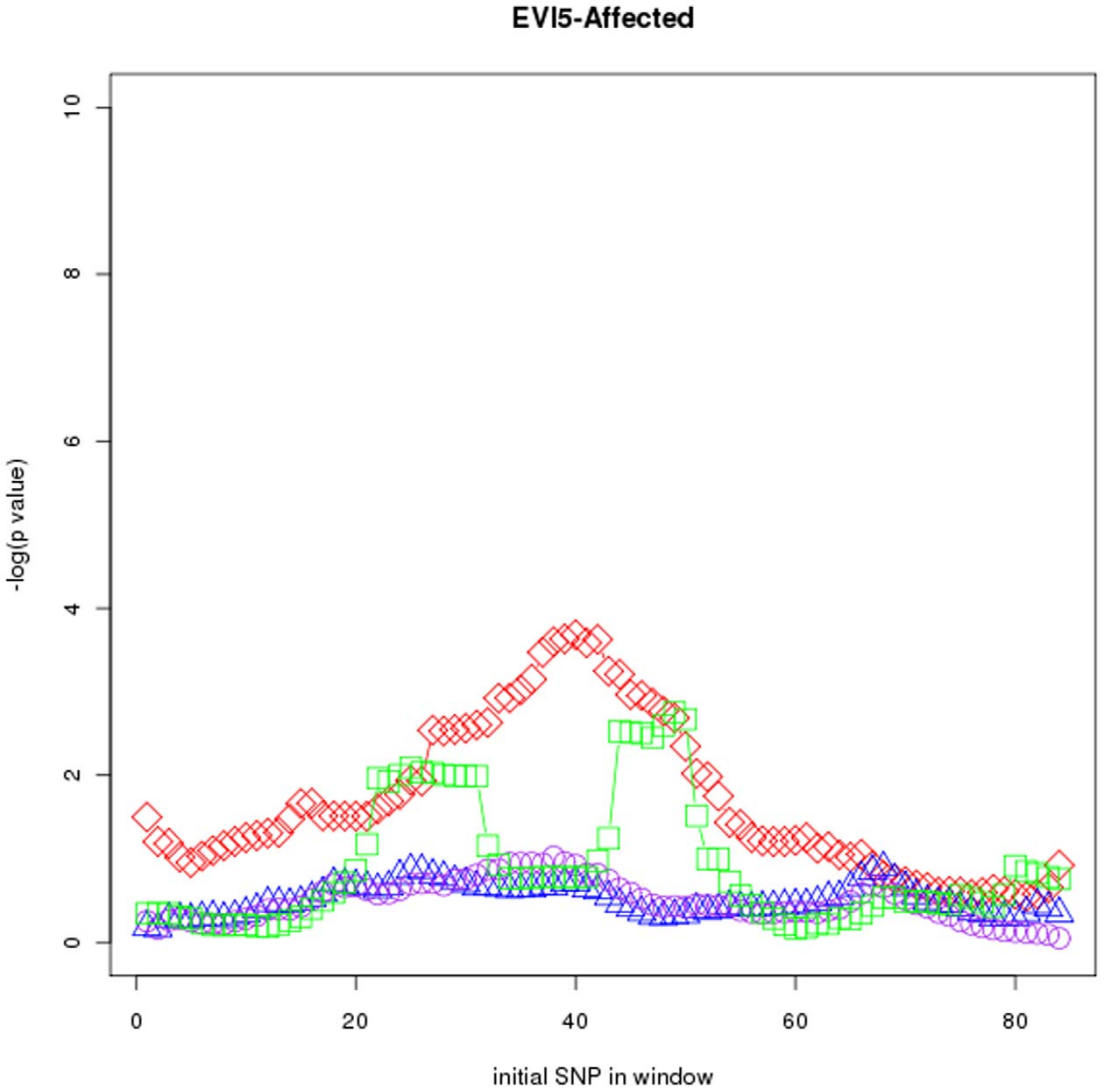
Sliding window maps for the 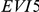-affected data set. Window size is 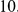 TDTs used were 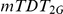 (red diamonds), 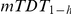 (green squares), 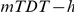 (purple circles) and 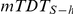 (blue triangles).

**Figure 6:**
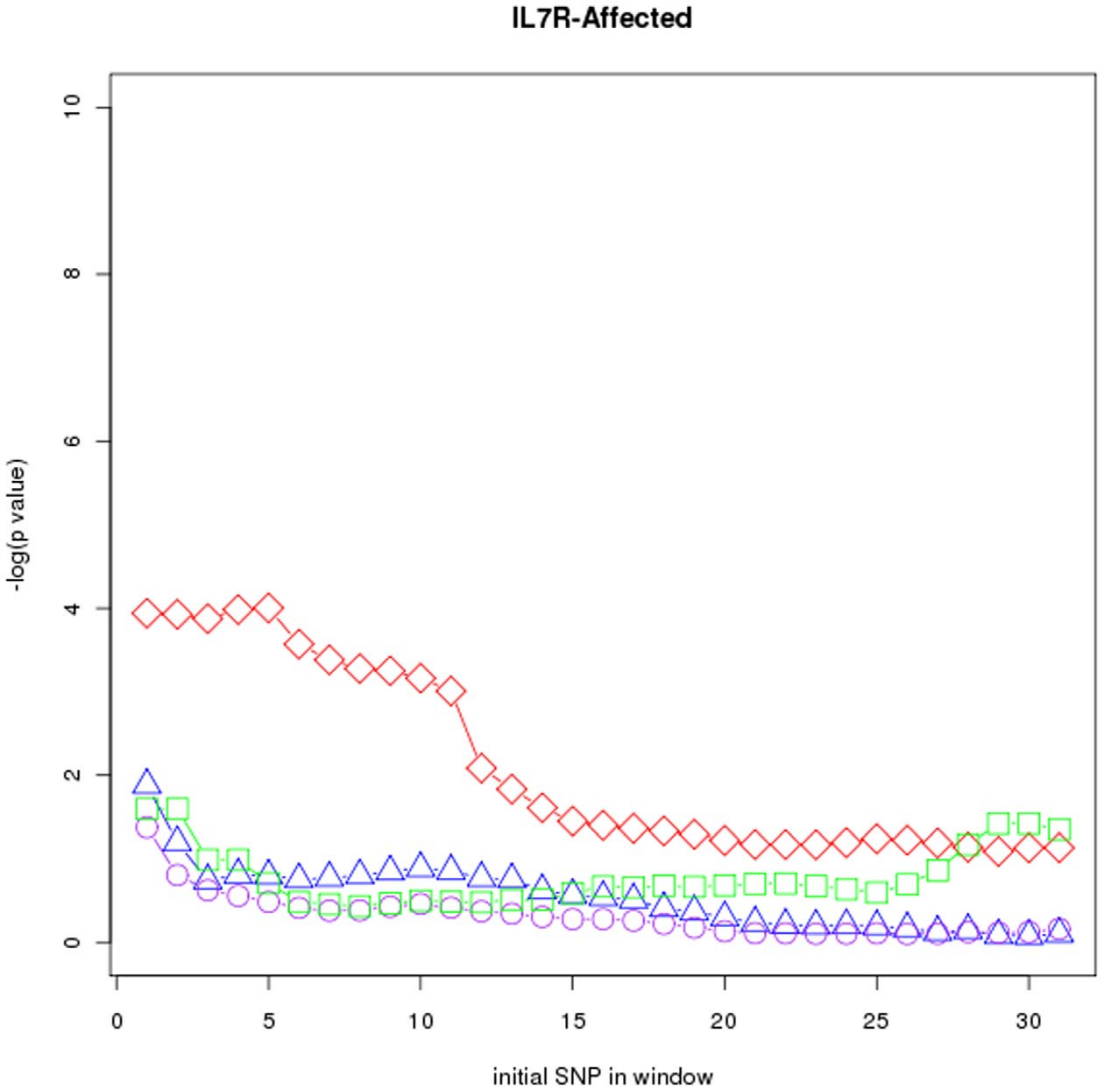
Sliding window maps for the 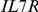-affected data set. Window size is 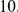 TDTs used were 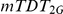 (red diamonds), 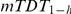 (green squares), 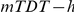 (purple circles) and 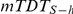 (blue triangles).

Figures 4, 5, and 6 respectively show p values for the MS IL2R-affected (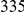 SNPs), MS EVI5-affected (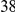 SNPs) and MS 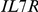-affected (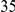 SNPs) data sets and windows of size 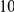. Genetic determinants of susceptibility to MS are complex, and until recently the only validated MS-associated polymorphic variants were found in the major histocompatibility complex (MHC) region [22]. Since 2007, the implementation of GWAS in combination with high-powered patient-control cohorts has completely changed this picture. Several GWAS and candidate gene studies have revealed the existence of non-MHC MS susceptibility loci of moderate genetic effect, and some of these including 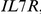 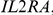 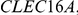 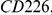 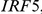 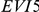 and 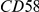 have been validated successfully in independent studies [23]–[30]. However, except for 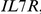 the causal SNP of the new determined risk loci are unknown. It is interesting to observe that the most significant associations found by 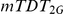 at the 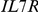 locus contained the rs6897932 SNP (SNP number 9), the causal variant of the association. For the IL2RA we have analyzed a wide region of the locus including the variants that have been associated to the MS and type 1 Diabetes (T1D). The most significant associations found by 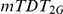 are located at the 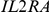 gene and 5 region of the gene, where the maximal association have been observed in MS and T1D studies [31].

Power and locus specificity are clearly higher in 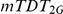 in these three data sets. Moreover, locus specificity is in general higher for 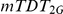 than for 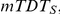 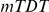 and 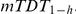 It seems that the alternative hypothesis built by 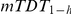 is in many cases too simplistic so that the more generic 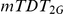 outperforms it. See Figures S46 to S51 (sliding windows) and S52 to S57 (CTDT maps) at the supplementary website (http://bios.ugr.es/2G) for results using different haplotype lengths 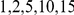 and 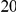 and all the data sets.

In agreement with the simulation results, in all cases a clear increase is detected in the superiority of 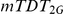 compared with the other multimarker TDTs used to detect power when window size increases. Although sample reproducibility of 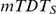 and 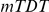 is very high when only one marker is used, in many cases only one marker is not enough to detect risk alleles. As an example, in MS 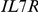-affected and MS 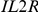-affected, associations found by these tests using only one or two markers lack in locus specificity and power (see Figures S46 and S47 at http://bios.ugr.es/2G) compared with results obtained by 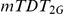 using more markers.

## Discussion


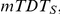
 and other tests alike, combine the segregation differences for each of the 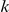 haplotypes in the form of summation of squared differences. 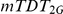 was derived by further relaxing the small assumptions made in the development of 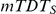 and 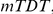 in order to reduce df. Thus, it does not assume any number of haplotypes 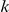 in the population and consider the whole effect of groups of haplotypes instead of considering the individual effect of each haplotype. Other tests compose groups in order to reduce df [2], [7], [11], [12]. However, 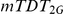 accomplishes this goal to its maximum: considering only two groups, regardless of the number of markers, means that df is always 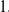 With this strong simplification we have shown that a highly significant way to collapse haplotype into two groups is in the way 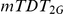 does: one group must represent disease susceptibility haplotypes and the other disease protective haplotypes. Therefore, we needed to collapse all the 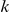 haplotypes in a sample (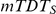 and 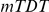 consider a unique but complex null hypothesis of no association for exactly those 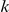 haplotypes, which is 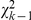 under the null) into only two groups. Moreover, for the test to be non-parametric, no assumptions could be made to set up the groups. Basically, we had to separate haplotypes into two groups using information from the sample, and not any prior knowledge we may have about the population. However, to avoid sample selection and therefore model overfitting, the groups had to be obtained from a different sample than the one used to compute the statistic. 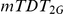 uses the simple holdout multisampling approach so that the sample is divided into two equally-sized data subsets. Simulation results showed the importance of having low df. Therefore, the fact that 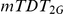 is asymptotically 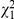 under the null hypothesis of no linkage, regardless of the number of markers, and thus the number of haplotypes, explains why it is on average more powerful than 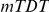 and 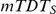 when tested in a second data set. The more generic hypothesis than the one built by 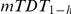 while keeping df to 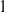 explains why it also outperforms 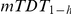 in simulations and in most real data sets. This hypothesis allows considering more than one disease variant or the situation in which the causal locus is not sequenced but markers in LD with it, so that more than one haplotype may be non recombinant haplotypes with the disease variant. Therefore, 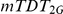 benefits from the use of long haplotypes to capture marker dependencies without reducing sample reproducibility due to sparse data.

Fine-mapping association may be performed by algorithms measuring differences in evolutionary haplotype trees [12], [13], [32]. These algorithms may strongly benefit analysis whenever 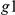 and 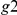 are used as starting point, instead of case versus control subsamples [12], [32], or transmitted versus nontransmitted subsamples [13].

Moreover, using the holdout approach seems to be an interesting solution that has also been applied to other group-based measures, such as 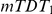 or to the more classic 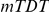 and 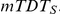 In contrast to the Bonferroni correction, which over-corrects the measure by performing a linear correction of p values, or other more complex and low accurate solutions, the holdout approach in 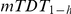 and 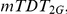 guarantees an asymptotically 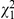 null distribution. Moreover, as the number of markers increases, validity of 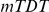 and 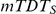 decreases and the holdout approach is a computationally feasible solution for genome-wide scan, compared with highly time-consuming simulation tests. Therefore, 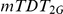 is a very competitive test to perform genome-wide scan because of its high performance in power, locus specificity, sample reproducibility and low computational cost.

In conclusion, we expect that 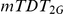 will prove useful in detecting association for any complex disease in which relative risk due to a disease locus can be low, and power needs to be maximized by using several markers at a time, without results being affected by sparse data. We also expect the two haplotype groups 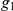 and 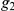 defined by 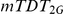 may also be used as the starting point for any method developed to perform haplotype fine mapping. Moreover, the test may be used as a first loci-selection step in the process of building more accurate genetic models to predict individual predisposition to complex diseases.

## Materials and Methods

In this section we explain which other tests were used to compare the performance of 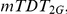 as well as the simulation and real data set studies performed for the comparisons.

A supplementary website has been created for this work at http://bios.ugr.es/2G, where Figures S1–S57, data sets, the software used to obtain the samples upon which the simulations were performed (scripts for linux and software in c++) and 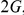 the software used to implement the method, are available.

### Comparative studies

We compared the performance in the state-of-the-art 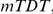 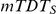 and 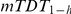 with 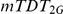 in both simulations and real data sets.

We chose these tests after comparing power and locus specificity among different state-of-the-art multimarker TDTs: 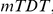 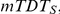 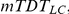 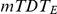 and 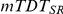 (data not shown). 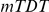 and 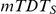 showed much higher power and locus specificity than the others and have a low computational complexity so that they are a practical choice for genome-wide scan.

We performed four different simulation studies: (1) We tested sample reproducibility in 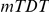 and 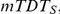 and observed a lack of it which increased with the number of markers. (2) We tested robustness to population stratification of 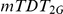 and 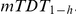 (3) We chose the holdout approach for all the tests to make sure power will be kept when testing on a second data set and therefore we compared power and locus specificity of 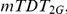 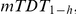 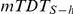 (the holdout version of 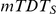) and 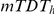 (the holdout version of 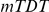), in a first data set and in a second data set to measure sample reproducibility. (4) We tested robustness of 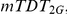 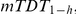 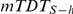 and 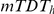 to different proportions of missing haplotypes.

After the simulation studies, we used real data sets and the holdout approach in order to guarantee that the results would be reproducible in a different independent data set, for all the multimarker TDTs used in the simulations.

### Simulation studies

Simulation analyses were performed using haplotype data sets of family trios. Simulations were similar to those used in several works [2], [5], [32], with the intention of evaluating both robustness to stratification population and sensitivity to a disease susceptibility locus. However we also added simulations to test locus specificity and sample reproducibility, as it above explained.

As one of our main goals was to have a useful test to perform genome-wide association filtering, computational complexity was a main issue and a linear relationship between computational complexity and the number of SNPs was highly desirable. Therefore, we applied the tests in a very feasible way in which only consecutive or overlapping clusters of SNPs (known as sliding windows) were tested together.

In order to simulate a cluster, as suggested by [33], we assumed that recombination rates between all markers tested were very low, which is equivalent to assuming they belong to the same low recombination block [34]. The recombination fraction within blocks (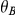) for a common population with exponential growing, such as an African population, has been estimated to be 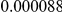 [35], and this is the value used in this work. By testing only consecutive SNPs at high LD we chose a method that is easily adaptable for use with genome-wide genotype data sets by using sliding windows. A disease susceptibility locus was placed at one extreme of the low recombination block. In those tests where the distribution under the null hypothesis is not known, statistical significance levels were obtained by using a permutation procedure known as the Monte Carlo test [16]. To investigate the effect of haplotype width, simulations were performed over different haplotype lengths within the low recombination block: 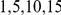 and 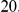


We also altered the way disease mutations were introduced, and decided to use the more realistic and now standard coalescent approach [36]. Thus, instead of considering only one ancestral chromosome with the disease causing mutation, or the improvement of using two ancestral chromosomes [5], a more realistic simulation of complex disease inheritance was used, in which the number of disease ancestral chromosomes can change according to the coalescent model, as any other gene does. We used MS sample to draw the populations [36].

Populations were drawn using msHOT [37], a program for generating samples based on the coalescent model that incorporates recombination. The samples for all the populations were obtained using *trioSampling*, a computer program we developed and which is available at the supplementary website.

Specific configurations required to test robustness, power and locus specificity are explained in the next subsections. A more detailed explanation of the simulations performed can be accessed at the supplementary website.

#### Robustness to population stratification

Type I error rates under population stratification and admixture were estimated based upon 1000 replications of the simulations here described. The data sets obtained from the populations were composed of 500 nuclear families with only one child (affected). In order to check whether 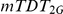 and 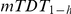 were robust to population stratification, we checked Type I errors in samples with affected individuals, for regions not in linkage with the disease locus (recombination fraction from the markers to the disease locus 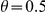), considering the simulation design of [2]. Therefore each stratified population drawn consisted of two sub-populations [2], with 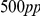 nuclear families from the first population and 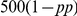 nuclear families from the second one, where 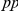 is the proportion of trios chosen from the first subpopulation. Populations were generated as described by [2] and [5], with founder haplotypes randomly having alleles at every marker independently. MAFs of 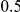 for the first subpopulation were assumed, while MAFs for the second subpopulation 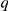 were parameterizable, with 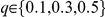. Frequencies for the disease allele at disease susceptibility locus (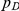) were 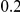 and 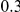 for the first and second subpopulation respectively. Families were randomly sampled by choosing haplotypes with the disease mutation with probability 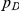 for the parents and randomly choosing the haplotypes transmitted to children considering recombinations. As it was done by [2] and [5], we also varied 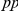 to have values 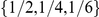. Therefore, by varying 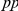 and 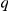, nine different scenarios where considered in order to test robustness. The samples obtained from each population were composed of 500 nuclear families with only one child.

#### Power and locus specificity

Association rates were estimated based upon 100 replications of the simulations here described. The data sets obtained from the populations were composed of 250 nuclear families with only one child (affected). When only one disease susceptibility locus was used, it was placed at one extreme of the low recombination block the markers belonged to. When two disease loci were used, the first was placed in the same way, while the second was placed at a position with 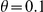 from that block, in order to model a second disease locus not in linkage with the tested markers [5]. The power of the tests was analyzed under three genetic models for one disease susceptibility locus: additive, dominant and recessive, and six genetic models for two disease susceptibility loci: additive, dom-and-dom, rec-or-rec, dom-or-dom, threshold and modified [5]. Different relative genotype risks 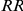 were also used: 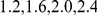 and 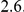 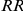 is defined as 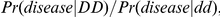 with 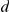 being the normal allele and 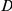 the disease allele for simulations with only one disease locus, and as 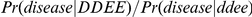 for simulations of two disease loci, with 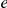 being the normal allele at the second disease locus and 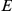 the disease allele at that locus [5]. Relative risks for all other genotypes were computed based on RR [5], [38] (see Table S1 on the supplementary website). To simulate a complex disease, disease loci were chosen among markers with MAFs in the intervals 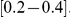


Simulations for power (sensitivity), i.e., assuming no recombination between the disease susceptibility locus and the markers tested, were similar to those used in several studies assuming one founder disease haplotype [2], [5], [32], except that SNPs tested together were assumed to be in high LD, i.e., they belong to the same low-recombination block [34].

To test locus specificity, we added six other different recombination fractions (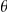) from the markers to the disease susceptibility locus, to the perfect LD (no recombination) used to test power: 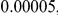 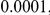 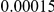 and 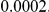


#### Sample reproducibility

To check sample reproducibility, for each data set used as a first step, a second independent data set from the same population with 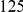 family trios as well was used to compute p values. The length similarity measure was used by all the tests to plug the second data set into the model learned from the first data set. Association rates using 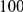 simulations were used to evaluate results.

#### Missing data

To check whether the tests were robust to missing data, we randomly chose a marker and a parent and deleted the parental genotype at that marker until reaching the desired proportion of missing data (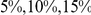 and 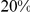).

### Real data

#### Sample reproducibility

Nine data sets of genotypes from trio families were used; one with offspring having Crohn's disease, the other nine with offspring having MS disease. The Crohn affected data set (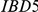-affected) is a publicly available set that was originally used by [39]. It consists of the genotype data of 103 SNPs typed in 129 trios with offspring having Crohn's disease [34]. The phenotype is the presence/absence of Crohn disease. The SNPs span across 500 kilobases at the 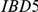/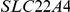 locus (5q31), and the region contains 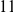 known genes. For MS disease, genotype information was obtained from a GWAS performed by the International Multiple Sclerosis Genetic Consortium. A DNA microarray (GeneChip Human Mapping 500 K Array Set, Affymetrix) was used by that study to examine 334,923 common genetic variants in 931 family trios, consisting of a patient with MS and both parents [23]. Nine regions corresponding to risk loci for MS as previously determined in well powered studies [23], [29] were chosen. Table 6 details information about the MS data sets. Results shown are meant to be highly valid and sample reproducible. Therefore, we chose the holdout approach in all the tests used. This way we increase the chances of finding similar power and locus specificity results if a second data set from the same population were to be used.

**Table 6:** Markers used in real data sets (affected and unaffected) for MS disease.

Data set	ch.	first SNP	last SNP	SNPs
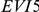	1	92388330	93651891	93
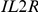	10	6103680	7715013	353
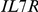	5	35847586	35991293	31
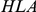	6	30736061	33163225	468
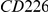	18	65550188	65997985	38
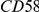	1	116677600	116983610	19
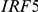	7	128055671	128309250	15
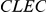	16	10947194	12685795	305

At first and last SNPs columns, the physical SNP position (NCBI build 36) is provided.

#### Data sets to test specificity

To check specificity in real data, for each data set with affected offspring we fabricated data sets for healthy trios, using data publicly available on the IHMP website [20], comprised of genotype data for 30 family trios (HapMap Phase II) typed in a population of Utah residents with ancestry from northern and western Europe (CEPH).

In the particular case of the 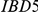-affected data set, most SNPs were not genotyped by the IHMP. As a solution, the 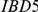-unaffected data set was composed by choosing the CEPH genotypes of only 656 consecutive SNPs (positions 276117 to 890934) out of 247,632 SNPs from chromosome 5, to correspond to the same region as in the 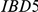-affected data set. It has to be noted that SNP density in the CEPH data set is about 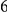 times higher than that in the 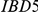-affected data set. To prevent differences in densities to bias results, we chose only one SNP for each cluster of 6 consecutive SNPs in the CEPH data set, so that only 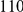 SNPs were selected to create the 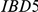-unaffected data set. 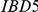-affected and 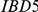-unaffected data sets are both available at the supplementary website. As it was done with disease data sets, data were split in order to test specificity in a second sample.

All unaffected data sets used to test specificity in MS samples are also available at the supplementary website.

#### Genome-wide exploration

In general, for a multimarker TDT to be used in data sets with genotypes spanning many bases, some techniques must be used to divide the region into smaller sequences so that individual tests can be applied to each sequence within a feasible computation time. In order to use a TDT to perform genome-wide pre-filtering, we only tested together SNPs in strong LD. Strategies to perform genome-wide searches using SNPs not in LD are very time consuming if they are exhaustive. For example, with only 2 markers tested together, computational time is quadratic to the number of markers. Thus, this is usually the maximum number of SNPs tested together in TDT or case/control studies that consider SNPs not in LD [40]. Therefore, we only considered consecutive sequences of SNPs to be tested together. Different haplotype lengths 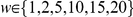 were used to investigate the effects upon power. To reduce random errors, we used sliding windows [34] of width 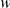 and an offset of 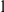 SNP. Before these calculations, we investigated dividing the whole chromosome into blocks of low recombination by using several algorithms proposed in the literature [41]. However, as blocks turned out to be very different depending upon the algorithm used (results not shown), we decided not to perform this division to avoid biased results.

#### Unknown haplotypes

If genotypes, instead of haplotypes, were the only information available, the phase for each family and marker was inferred using information from the family [5], [42]. Phase for those markers that remained unsolved, was estimated by using the E-M algorithm under the restriction of family information [2], [5], [43]. Other algorithms for phase resolution are known to be more accurate but at a high computational cost, such as Phase [44], an algorithm that uses Gibbs sampling for phase reconstruction of each individual.

For 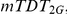 each data set of genotypes was divides into two equal-size data subsets, from which haplotypes were obtained.

#### Comparative TDT maps

For a quick visual comparison of power and specificity between these different measures, we also used CTDT maps [21] for all the data sets and all the window sizes used. These maps are colored only in those regions found in association. Results from each TDT are plotted in a different pair of consecutive rows. The first row in a pair (white color background) shows results from the affected data sets at every marker to test power. The second row in the pair (gray background) shows results from the unaffected data sets to test specificity at every marker. All rows in a map have the same length, as it represents the number of markers in the sample. The height of a row represents the association level. If height is 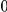 when used with affected offspring, it means that the p value at that marker is larger than 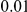, and the test is considered powerless to detect association to that SNP. When used with samples of unaffected offspring, height must be 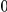 except in the situation of a protective locus.

## Supporting Information

Text S1
**An Appendix which shows that** 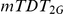 **follows a** 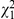 **distribution under the null hypoyhesis of no linkage.**
(PDF)
