# Supplementary material for: Sample Reproducibility of Genetic Association Using Different Multimarker TDTs in Genome-Wide Association Studies: Characterization and a New Approach
Source: PLoS One. 2012 Feb 17;7(2):e29613. doi: 10.1371/journal.pone.0029613 (PMC3281822; doi:10.1371/journal.pone.0029613)
Supplement: Text S1 — An Appendix which shows that follows a distribution under the null hypoyhesis of no linkage. (PDF) [file pone.0029613.s001.pdf]

Supporting Information Text S1 to *Sample Reproducibility of Genetic Association using Different Multimarker TDTs in Genome-wide Association Studies: Characterization and a New Approach*

María M. Abad-Grau[1,*], Nuria Medina-Medina[1], Rosana Montes-Soldado[1], Fuencisla Matesanz[2], Vineet Bafna[3]
1 Departamento de Lenguajes y Sistemas Informáticos, ETS Ingeniería Informática y de Telecomunicaciones - CITIC, Universidad de Granada, Granada, Spain
2 Instituto de Parasitología y Biomedicina López Neyra, Consejo Superior de Investigaciones Científicas, Granada, Spain
3 Department of Computer Science and Engineering, University of California, San Diego, La Jolla, CA, USA
∗ E-mail: mabad@ugr.es

Appendix

$mTDT_{2G}$ uses a subset S_1 with half of the trios to build the groups g_1 and g_2 and the other half S_2 to compute the statistic.

Let N_{ij} be the count in S_1 of heterozygous parents with haplotypes i, j transmitting haplotype i to their child, and N_{ji} be the count in S_1 of parents with the same genotype but transmitting haplotype j to their child. Let consider N_{ij} a realization of the random variable X_{ij}, $i = 1, \ldots, k$, $j = i + 1, \ldots, k$, and N_{ji} a realization of the random variable X_{ji} and N_{ii} a realization for S_1 of the random variable X_{ii}. Thus, $X_{ji} = N_{ij} - X_{ij}$ holds.

Let $n_{iT} = \sum_{j \neq i} N_{ij}$ and $n_{iU} = \sum_{j \neq i} N_{ji}$ realizations for S_1 of the random variables $X_{iT} = \sum_{j \neq i} X_{ij}$ and $X_{iU} = \sum_{j \neq i} X_{ji}$ respectively. Under the null hypothesis of no linkage, every heterozygous parent has the same probability of transmitting one haplotype or the other to their offspring. Therefore, X_{ij} and X_{ji} are respectively binomial with parameters $(n_{ij}, 1/2)$ and $(n_{ji}, 1/2)$ and X_{iT} and X_{iU} are respectively binomial with parameters $(n_{iT}, 1/2)$ and $(n_{iU} = n_i - n_{iT}, 1/2)$ with n_i being defined as $n_{iT} + n_{iU}$. Hence, when using S_1 to build the groups, it turns out that every haplotype i in S_1 has the same probability of belonging to groups g_1 and g_2.

Once the two groups are defined by using S_1, only those heterozygous parents in S_2 with one haplotype in each group are used in $mTDT_{2G}$. In the case a haplotype in S_2 does not exist in S_1, the haplotype will be considered as belonging to the group where the most similar haplotype in S_1 was placed. As it was said above, the similarity between haplotypes in $mTDT_{2G}$ is computed by the length similarity measure [?, ?, ?]. Let n'_{ij} be the counts of heterozygous parents in S_2 with either $h_i \in g_1$ and $h_j \in g_2$ or the other way around. Under the null hypothesis, N'_{ij}, the count of heterozygous parents in S_2 with either $h_i \in g_1$ and $h_j \in g_2$ or the other way around transmitting haplotype i to their offspring is a realization of a random variable X'_{ij} which is binomial with

parameters $(n'_{ij}, 1/2)$ and in an equivalent manner the count of those parents in S_2 transmitting haplotype j N'_{ji} is a realization of a random variable X'_{ji} which also is asymptotically binomial with parameters $(n'_{ij}, 1/2)$.

Therefore, the total counts of heterozygous parental genotypes with one haplotype in each group with the haplotype in g_1 being transmitted to the offpsring $n_{g_1g_2}$ and with the haplotype in g_2 being transmitted to the offspring $n_{g_2g_1}$ are then realizations of the random variables

$$X_{g_1g_2} = \sum_{h_i \in g_1, h_j \in g_2} X'_{ij}$$

and

$$X_{g_2g_1} = \sum_{h_i \in g_1, h_j \in g_2} X'_{ji}$$

As only those heterozygous parents with one haplotype in each group are used by $mTDT_{2G}$, is straightforward to show that $mTDT_{2G}$ may be considered a generalization of the simple biallelic monomarker TDT, a McNemar test, in which instead of two haplotypes it uses two groups of haplotypes and considers all the haplotypes in the same group as the same one so that parents with both haplotypes in the same group are disregarded.

Therefore, the variance of $mTDT_{2G}$ is

$$V_{mTDT_{2G}} = Var(T_{2G}) = Var\left[\frac{(X_{g_1g_2} - X_{g_2g_1})^2}{X_{g_1g_2} + X_{g_2g_1}}\right]$$

The random variable T_{2G} can be rewritten as:

$$T_{2G} = \frac{\left[\sum_{h_i \in g_1}\left[\sum_{h_j \in g_2}(2X'_{ij} - n'_{ij})\right]\right]^2}{\sum_{h_i \in g_1}\sum_{h_j \in g_2} n'_{ij}} = \frac{\left[\sum_{h_i \in g_1}\left[\sum_{h_j \in g_2} 2X'_{ij} - n'_i\right]\right]^2}{\sum_{h_i \in g_1} n'_i},$$

with n'_i being defined only for haplotypes in g_1 as $\sum_{h_j \in g_2} n'_{ij}$.

Thus,

$$T_{2G} = \frac{\left[\sum_{h_i \in g_1, h_j \in g_2} 2X'_{ij} - n_g\right]^2}{n_g}$$

is asymptotically χ^2_1 $(Var(T_{2G}) = 2)$ under the null hypothesis, as its square root is a standard normal, if we take into account that under the null X'_{ij} is binomial with parameters $(n'_{ij}, 1/2)$, for large samples $2X'_{ij}$ is asymptotically normal with mean and variance n'_{ij} and hence, as X'_{ij} and X'_{ik} are independent for any $k \neq j$, $\sum_{h_i \in g_1, h_j \in g_2} 2X'_{ij} - n'_i$ is normal with variance n'_i, and $\sum_{h_i \in g_1, h_j \in g_2} 2X'_{ij} - n_g$ is normal with variance n_g. $\square$
